# Loss of FMRP Impaired Hippocampal Long-Term Plasticity and Spatial Learning in Rats

**DOI:** 10.3389/fnmol.2017.00269

**Published:** 2017-08-28

**Authors:** Yonglu Tian, Chaojuan Yang, Shujiang Shang, Yijun Cai, Xiaofei Deng, Jian Zhang, Feng Shao, Desheng Zhu, Yunbo Liu, Guiquan Chen, Jing Liang, Qiang Sun, Zilong Qiu, Chen Zhang

**Affiliations:** ^1^State Key Laboratory of Membrane Biology, School of Life Sciences, Peking University-IDG/McGovern Institute for Brain Research, Peking University Beijing, China; ^2^Peking-Tsinghua Center for Life Sciences, Academy for Advanced Interdisciplinary Studies, Peking University Beijing, China; ^3^CAS Key Laboratory of Primate Neurobiology, Institute of Neuroscience, Chinese Academy of Sciences Shanghai, China; ^4^Key Laboratory of Mental Health, Institute of Psychology, Chinese Academy of Sciences Beijing, China; ^5^Department of Psychology, Peking University Beijing, China; ^6^Institute of Laboratory Animal Science, Peking Union Medical College/Chinese Academy of Medical Sciences Beijing, China; ^7^MOE Key Laboratory of Model Animal for Disease Study, Model Animal Research Center, Nanjing University Nanjing, China; ^8^Key Laboratory for Neuroscience, Ministry of Education/National Health and Family Planning Commission, Peking University Beijing, China

**Keywords:** FXS, hippocampus, long-term plasticity, spatial learning, intellectual disability

## Abstract

Fragile X syndrome (FXS) is a neurodevelopmental disorder caused by mutations in the *FMR1* gene that inactivate expression of the gene product, the fragile X mental retardation 1 protein (FMRP). In this study, we used clustered regularly interspaced short palindromic repeats (CRISPR)/CRISPR-associated protein 9 (Cas9) technology to generate *Fmr1* knockout (KO) rats by disruption of the fourth exon of the *Fmr1* gene. Western blotting analysis confirmed that the FMRP was absent from the brains of the *Fmr1* KO rats (*Fmr1^exon4-KO^*). Electrophysiological analysis revealed that the theta-burst stimulation (TBS)–induced long-term potentiation (LTP) and the low-frequency stimulus (LFS)–induced long-term depression (LTD) were decreased in the hippocampal Schaffer collateral pathway of the *Fmr1^exon4-KO^* rats. Short-term plasticity, measured as the paired-pulse ratio, remained normal in the KO rats. The synaptic strength mediated by the α-amino-3-hydroxy-5-methyl-4-isoxazolepropionic acid receptor (AMPAR) was also impaired. Consistent with previous reports, the *Fmr1^exon4-KO^* rats demonstrated an enhanced 3,5-dihydroxyphenylglycine (DHPG)–induced LTD in the present study, and this enhancement is insensitive to protein translation. In addition, the *Fmr1^exon4-KO^* rats showed deficits in the probe trial in the Morris water maze test. These results demonstrate that deletion of the *Fmr1* gene in rats specifically impairs long-term synaptic plasticity and hippocampus-dependent learning in a manner resembling the key symptoms of FXS. Furthermore, the *Fmr1^exon4-KO^* rats displayed impaired social interaction and macroorchidism, the results consistent with those observed in patients with FXS. Thus, *Fmr1^exon4-KO^* rats constitute a novel rat model of FXS that complements existing mouse models.

## Introduction

Fragile X syndrome (FXS) is the most common heritable cause of mental retardation and intellectual disability in humans (Pieretti et al., 1991). The prevalence of FXS is about 1 in 4,000 men and 1 in 6,000–8,000 women (de Vries et al., 1997). Approximately 85% of male and 25% of female patients with FXS show significant intellectual and developmental disability (Lozano et al., 2016). Fragile X mental retardation protein (FMRP) is enriched in the brain and testes (Devys et al., 1993; Bakker et al., 2000), in accordance with the mental retardation and macroorchidism exhibited by most patients with FXS (Hagerman, 1987; Martin and Arici, 2008; Saldarriaga et al., 2014).

The most studied FXS animal model is the *Fmr1* knockout (KO) mouse, which is generated by disrupting either exon 5 (The Dutch-Belgian Fragile X Consortium, 1994) or exon 1 and the promoter region (Mientjes et al., 2006) of the *Fmr1* gene. Both *Fmr1* KO mouse lines lack FMRP in the brain and show diverse behavioral phenotypes and synaptic physiology deficits, some of which recapitulate the clinical symptoms of patients with FXS [reviewed in (Kazdoba et al., 2014)]. *Fmr1* KO mice with transgenic expression of the human *FMR1* gene have demonstrated reduced anxiety and increased exploratory behavior in addition to the correction of some KO behavior phenotypes (Peier et al., 2000; Spencer et al., 2008). Mouse models with expansion of the CGG trinucleotide repeat in the *Fmr1* gene have also been developed to mimic the genetic changes observed in humans with FXS (Bontekoe et al., 2001). However, mouse FXS models have yielded mixed results or failed to reproduce several core FXS clinical phenotypes, including global cognitive dysfunction. For example, *Fmr1* KO mice showed normal behavior in the probe trial in the Morris water maze test, with the exception of a subtle change during the reversal trial when the platform was changed to the opposite position (The Dutch-Belgian Fragile X Consortium, 1994; Kooy et al., 1996). In the radial arm maze test, *Fmr1* KO mice exhibited a normal working memory in comparison with that of the wild-type (WT) mice (Yan et al., 2004). Long-term potentiation (LTP) is a major type of long-lasting synaptic plasticity and is associated with learning and memory. Protein synthesis-dependent late-phase LTP in the hippocampus of *Fmr1* KO mice is still controversial (Hu et al., 2008; Shang et al., 2009; Koga et al., 2015).

Rats are genetically more similar than mice to humans. The usage of rats in scientific research began in the middle of the 19th century (Baker et al., 1979). Rats are widely used in studies of neurological disorders, such as epilepsy, depression, Parkinson’s disease, stroke, and vascular brain disorders (Kerkerian-Le Goff et al., 2009; Melani et al., 2010; Bailey et al., 2011; Nabika et al., 2012; Tayebati et al., 2012; Liao et al., 2013; Russo et al., 2013). Inactivation of the *Fmr1* gene in rats via zinc finger nuclease (ZFN) technology targeting of the junction region between intron 7 and exon 8 was recently reported (Hamilton et al., 2014) in a three-chamber test in which 21 amino acids were deleted from the FMRP. This line of KO rats (*Fmr1^exon8-KO^*) displayed social dysfunction, an autism-related phenotype. Further studies of *Fmr1^exon8-KO^* rats revealed abnormal neuronal morphology in the superior olivary complex and impaired sound processing (Engineer et al., 2014; Ruby et al., 2015), as well as increased metabotropic glutamate receptor (mGluR)-dependent hippocampal long-term depression (Till et al., 2015). Juvenile *Fmr1^exon8-KO^* rats showed dysfunction in regulating the circuit state in the visual cortex (Berzhanskaya et al., 2016, 2017).

Fragile X mental retardation 1 protein is highly expressed in neurons, and dysregulation of FMRP causes impairment of synaptic strength and neural circuit development. In the present study, a KO rat model was generated by specifically targeting exon 4 using clustered regularly interspaced short palindromic repeats (CRISPR)/CRISPR-associated protein 9 (Cas9) technology, ensuring that regions downstream of exon 4, including the full RNA binding sequence, were not translated. We examine the physiology in hippocampal CA1 pyramidal neurons of the *Fmr1^exon4-KO^* rat. Loss of FMRP can lead to deficits in basal synaptic transmission and long-term synaptic plasticity, including theta burst stimulation (TBS)–induced LTP, a low-frequency stimulus (LFS)–induced long-term depression (LTD), and a 3,5-dihydroxyphenylglycine (DHPG)–induced LTD in the *Fmr1^exon4-KO^* rat. The knockout (KO) *Fmr1* gene in rats also contributes to abnormal cognitive behaviors.

## Materials and Methods

### Animals

*Fmr1* KO rats were produced by the CRISPR/Cas9 method and maintained in the laboratory animal center of Peking University. This line was created via the outbred Sprague-Dawley background. The KO rat lines were maintained with heterozygous female and WT male breeding pairs. The genotypes of the animals were identified. KO and WT rats aged 8–12 weeks were used in the study. The rats were kept in a temperature- and relative-humidity-controlled environment (22 ± 2°C, 40–70%) with a 12-h light/dark cycle and free access to food and water. All animal studies were conducted in accordance with the *Guide for the Care and Use of Laboratory Animals* (8th edition) and approved by the Institutional Animal Care and Use Committee of Peking University. All tests were performed using WT and KO littermates derived from breeding heterozygous female rats with Sprague-Dawley WT male rats. All behavioral tests were conducted in a temperature-controlled (24 ± 2°C) test room between 14:00 and 18:00. After each test, the apparatus and the test area were cleaned with 75% ethanol to remove olfactory cues. In all the behavioral assays, the light intensity was 15–20 lx, and the sound intensity was less than 60 dB. All the behavior tests and electrophysiological measurements were performed in a blinded manner.

### DNA Analysis and Genotyping

DNA was obtained from rat-toe tissue samples by incubation with 500 μg/mL proteinase K (Amresco, Solon, OH, United States) in 400 μL lysis buffer [10 mM Tris–HCl, 5 mM EDTA, 0.2% sodium dodecyl sulfate (SDS), 200 mM NaCl] for 6–8 h at 55°C. After incubation, 400 μL isopropyl alcohol was added to precipitate DNA. The suspensions were centrifuged at 13,000 rpm for 10 min, after which the supernatant was removed. Next, 1 mL of 70% ethanol was added to the sample, which was centrifuged at 13,000 rpm for 10 min, after which all ethanol was removed, and the tube was dried. The DNA was dissolved in 100 μL 5 mM Tris buffer (pH 8.0) for 30 min at 55°C. PCR genotyping was performed using 2× *Taq* PCR Mix (Aidlab, Beijing, China), *Fmr1* forward primer (5′-CCG TGA GTT CTC AAG TTG TTT CCA-3′), and *Fmr1* reverse primer (5′-GGG ATT AAG AGC ATG CAT CAC CAT-3′). Polymerase chain reaction (PCR) was performed with the following protocol on a MyCycler Thermal Cycler^TM^ (Bio-Rad, Hercules, CA, United States): 95°C for 4 min, 95°C for 30 s, 60°C for 30 s (lowered 0.5°C per cycle), 72°C for 30 min (30 cycles); 95°C for 30 s, 45 °C for 30 s, 72°C for 30 min (30 cycles); 72°C for 7 min, and a final hold at 4°C. PCR products were run on 1% agarose gel. The amplicon was approximately 500 bp. The amplicon was sequenced to determine the genotypes of the rats.

### Western Blot Analysis

The brains of the WT and *Fmr1* KO rats were homogenized in phosphate-buffered saline (PBS) containing 0.1 mM ethylene glycol-bis (β-aminoethyl ether)-N,N,N′,N′-tetraacetic acid (EGTA), 1 mM phenylmethylsulfonyl fluoride (PMSF), 1 μg/mL pepstatin, 1 μg/mL leupeptin, 2 μg/mL protinin, and 1% Triton X-100. Proteins in the homogenate were extracted for 2 h at 4°C, after which insoluble material was removed with centrifugation (1 h at 100,000 × *g*). Protein concentrations were determined using a BCA Protein Assay Kit (Thermo Scientific, Carlsbad, CA, United States). Each protein sample (100 μg) was boiled in sodium dodecyl sulfate (SDS)-loading buffer, subjected to electrophoresis on a 10% SDS-polyacrylamide gel, and electroblotted onto a nitrocellulose membrane as described previously (Wei et al., 2017). FMRP was detected using a rabbit antibody (Cell Signaling Technology, Danvers, MA, United States, #4317, 1:1,000) as the primary antibody. GAPDH was detected using a rabbit antibody (Abmart, Shanghai, China, P30008, 1:1,000) as the primary antibody. IRDye 800CW-labeled anti-rabbit IgG was used as the secondary antibody and was detected with an Odyssey Infrared Imager System (LI-COR, Lincoln, NE, United States).

### RNA Isolation and Quantitative RT-PCR

Total RNA was isolated from the hippocampus and cortex samples that were collected from three WT rats and three *Fmr1* KO rats at approximately 8–12 weeks of age. The samples were homogenized in a glass-Teflon^®^ homogenizer according to the protocol supplied with TRIzol^®^ Reagent (Life Technologies, Carlsbad, CA, United States). The concentration of RNA was measured with spectrophotometry. The reaction volume consisted of 2 μg of total RNA, 5× buffer (Takara, Kusatsu, Japan), Rt enzyme mix (Takara), oligo (dT) (Takara), Random6 primer (Takara), and RNase-free H_2_O (to a final volume of 20 μL). The amplification program was as follows: 37°C for 15 min, 85°C for 5 s, and a final hold at 4°C. Quantitative PCR was carried out in an MX 3000P^TM^ (Agilent Stratagene, Palo Alto, CA, United States) real-time PCR system with 2× SYBR Green qPCR Mix (Aidlab, PC3302) using designed primers. Three primer pairs were designed for the *Fmr1* amplicon: a pair crossing exons 1, 2, and 3 (forward primer: 5′-GGC TCC AAT GGC GCT TTC TA-3′; reverse primer: 5′-TAA CCT ACA GGT GGT GGG-3′); a pair crossing exons 4 and 5 (forward primer: 5′-TAA CCT ACA GGT GGT GGG-3′); reverse primer: 5′-TGT GAC AAT TTC ATT GTA TG-3′); and a pair crossing exons 7 and 8 (forward primer: 5′-GAA ATG AAG AAG CCA GTA A-3′; reverse primer: 5′-AAT CAA TAG CAG TGA CCC-3′). GAPDH was used as an internal control (forward primer: 5′-CCT GGA GAA ACC TGC CAA GTA T-3′; reverse primer: 5′-CCC TCA GAT GCC TGC TTC A-3′) (Mientjes et al., 2006). Relative expression levels were calculated using the 2^-Δ ΔCT^ method.

### Three-Chamber Sociability Test

The experiment was executed as described previously (Chung et al., 2015; Xu et al., 2015; Lo et al., 2016). WT (*n* = 8) and KO (*n* = 11) male rats aged 8–12 weeks were tested in a three-chamber apparatus (40 cm × 34 cm × 24 cm) with each side chamber connected to the middle chamber by a corridor (10 cm × 10 cm × 15 cm). Before the test day, the animals were allowed to habituate the environment for 60 min. At the beginning of the test, each rat was placed into the middle chamber and allowed to move freely through all three chambers for 5 min. For the sociability tested, a novel rat (stranger1) locked in a small cage was placed in one of the side chambers, and an empty cage of the same size and design was placed in the other side chamber. The test animal was monitored and allowed to explore both chambers for 10 min, and the total time spent in each chamber was measured. The intruder was randomly assigned to one of the side chambers to avoid a side bias. In the social novelty tested, a new unfamiliar rat (stranger2) was enclosed in the cage that had been empty during the sociability test. All model rats were male and were the same age as the testing rat but had no previous contact with each other. Data were analyzed with one-way analysis of variance (ANOVA), and a two-sided Student’s *t*-test was used to perform the preference index analysis.

### Assessment of Motor Activity Using a Force-Plate Actometer

A force-plate actometer (Bioanalytical Systems, West Lafayette, IN, United States) was used as an open field to evaluate hyperactivity and motor function. The actometer consisted of a Plexiglas^®^ enclosure (33 cm high), a 44 cm × 44 cm plate, four force transducers, and a recording and analysis system. The area was defined as the center point to 11.64 cm, and the outer area was defined as the zone from 11.64 to 44.00 cm. The animals were placed in a force-plate actometer chamber (44 cm × 44 cm) in a dark and sound-attenuating cabinet for 60 min. Data were collected and stored during time units of 40.96 frames, with a sampling frequency of 100 points/s. The distance traveled, the tremor index, focused stereotypy, bouts of low mobility (BLM; 10 s within a 20 mm radius), and time spent in the center field were recorded. The temperature of the test room was controlled (24 ± 2°C). Before the test, rats were allowed to adapt to the environment for 1 h.

### Morris Water Maze Assay

Tests were conducted in a circular black tank 150 cm in diameter containing 22 cm of water (24 ± 2°C). A circular platform (8 cm in diameter) was placed 2 cm beneath the water level. The swim paths of the rats were tracked, digitized, and stored for later behavioral analysis using Ethovision (Noldus, Wageningen, Netherlands). The water maze was divided into four quadrants (I, II, III, and IV). The rats were given four trials per day (30 min inter-trial intervals, ITIs) for four consecutive days during the spatial learning phase. During the learning phase, each animal was randomly placed in a different quadrant, with the exception of the quadrant where the platform was placed in each trial. The maximum trial length was 60 s. When a rat did not find the platform within 60 s, the latency time was calculated as 60 s. After the rats were taken out of the pool, they were dried with towels and returned to their cages. The platform was removed during the probe test. During the reverse training phase, the platform was placed in the third quadrant, which was opposite that used during the learning phase.

### Elevated Plus Maze

The elevated plus maze (EPM) was used to assess anxiety-like behavior. The black-painted maze consisted of four arms (50 cm length × 10 cm width). Two opposite open arms without walls and two opposite closed arms with 35 cm high walls formed a “+” shape. The maze was elevated 76 cm above the floor by four metal legs under each arm. Each rat was placed at the junction of the open and closed arms, facing an open arm. The rat was allowed to freely explore the entire maze for 5 min. The time spent in the open arms and closed arms were recorded using the Xeye Aba V3.2 tracking system.

### Slice Physiology

Hippocampal slices (400 μm) were produced from 8-week-old male WT and *Fmr1* KO rats as previously (Wei et al., 2016). Animals were anesthetized using pentobarbital (10 mg/mL, 0.1 mL/10 g) and euthanized via decapitation. The brain was quickly removed to an ice-cold dissection solution with a pH of 7.3–7.4. The solution contained 213 mM sucrose, 10 mM glucose, 3 mM KCl, 1 mM NaH_2_PO_4_, 0.5 mM CaCl_2_, 5 mM MgCl_2_, and 26 mM NaHCO_3_. Transverse slices were cut in ice-cold dissection solution on a vibrating blade microtome (Leica VT-1200s, Wetzlar, Germany). Slices were maintained for 1 h at room temperature in artificial cerebrospinal fluid (ACSF) containing the following: 10 mM glucose, 125 mM NaCl, 5 mM KCl, 2 mM NaH_2_PO_4_, 2.6 mM CaCl_2_, 1.3 mM MgCl_2_, and 26 mM NaHCO_3_ (pH 7.3–7.4). The ACSF and dissection solution were gassed with 95% O_2_ and 5% CO_2_. For the recordings, slices were individually transferred to the recording chamber and mounted on the stage of an upright microscope (Olympus BX51WI, Tokyo, Japan). The bathing solution was kept at room temperature and constantly exchanged through a gravity-driven perfusion system with a flow rate of approximately 2 mL/min during the experiment. Stimuli was delivered to the slice via a concentric bipolar electrode (CBBEB75, FHC, Bowdoin, ME, United States). Microelectrodes filled with ACSF (4–7 MΩ) were used to record field excitatory postsynaptic potentials (fEPSPs) from the stratum radiatum of the CA1 region. An EPC10 Patch Clamp Amplifier (HEKA, Lambrecht, Germany) was used to record fEPSPs, the values of which were calculated by measuring the onset (a 30–70% rising phase) slope of the fEPSP. TBSs were used to induce LTP as described previously (Zhang C. et al., 2009; Zhang et al., 2010). Each TBS was composed of five episodes of stimulation delivered at 0.1 Hz, whereas each episode contained 10 stimuli trains of five pulses (100 Hz) delivered at 5 Hz. The average response was expressed as a percentage of the pre-TBS response. A LTD was induced with low-frequency stimulation (1 Hz, 900 pulses) or DHPG (100 μM, 10 min, Tocris, Bristol, United Kingdom). Anisomycin (20 μM, MedChemExpress, Monmouth Junction, NJ, United States) was added to the ACSF 1 h before recording and throughout the recordings. The synaptic ratio was calculated as the percentage of the second fEPSP slope vs. the first slope in individual slices.

### Histology

Cresyl violet (Nissl) staining was used to evaluate the cytoarchitecture in the hippocampal regions. The rats were deeply anesthetized with tribromoethanol (240 mg/kg, Sigma–Aldrich, St. Louis, MO, United States) and transcardially with 4% paraformaldehyde (PFA) (w/v) in PBS. The brains were removed and dipped into fresh 4% PFA for an additional 48 to 72 h to be post-fixed at room temperature. Then the samples were embedded in paraffin and sectioned. Four-micron-thick sections were used for staining. The tissue slides were dried for 30 min at 55°C and then rewarmed at room temperature. The sections were washed at the time for distilled water and stained with a 0.5% Cresyl violet solution for 10 min. Then, the sections were washed again with distilled water, dehydrated in a graded ethanol series (95%, 1 min; 95%, 30 s; 100%, 1 min; and 100%, 1 min), and subsequently soaked three times in xylene, 5 min per time. Using the mounting medium, the sections were covered with a coverslip. Finally, an Axio Scan.Z1 (Zeiss, Oberkochen, Germany) digital slide scanner with an X20 objective was used for image acquisition.

## Results

### Generation of *Fmr1* KO Rats with CRISPR/Cas9-Mediated Genome Editing

**FIGURE 1:**
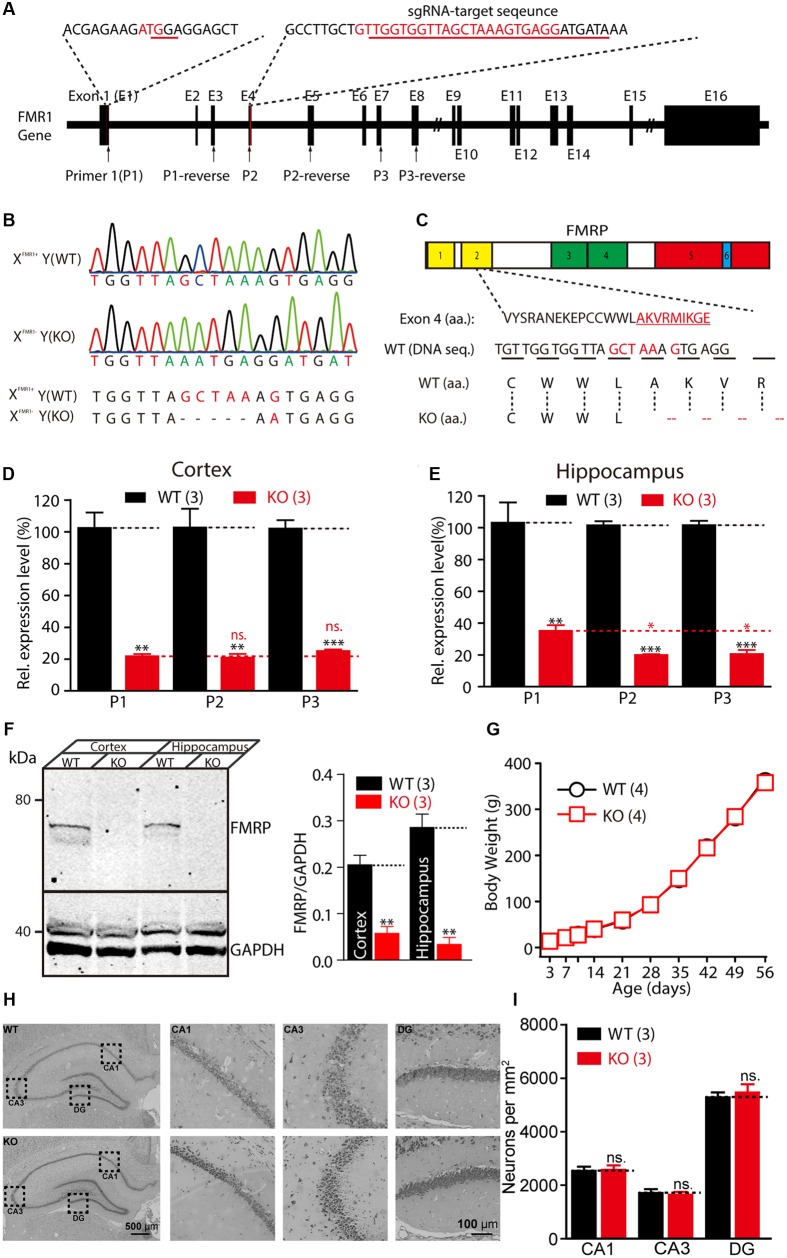
Generation of *Fmr1* knockout (KO) rats using the CRISPR/Cas9 method. **(A)** Targeting of the *Fmr1* gene. **(B)** Genotypes of the *Fmr1* KO rats were determined with sequencing of polymerase chain reaction (PCR) products amplified from tail DNA. **(C)** Localization of the deletion in FMRP. Domains in FMRP: Agenet-like 1 (no. 1, yellow), Agenet-like 2 (no. 2, yellow), KH 1 (no. 3, green), and KH 2 (no. 4, green), interaction with RANBP9 (no. 5, red) and RNA-binding RGG box (no. 6, blue). **(D,E)** Relative expression levels of *Fmr1* transcripts in the cerebral cortex **(D)** and hippocampus **(E)** were measured. Student’s *t*-test was used to compare the expression levels of the two groups. **(F)** *Fmr1* KO brain lacking expression of the fragile X mental retardation 1 protein (FMRP). Brain homogenates (100 μg) were subjected to western blot analysis (left). Normalized expression levels of FMRP in rat brain homogenate (right). **(G)** *Fmr1* KO rats showed a normal developmental curve. **(H)** Hippocampal regions stained with Nissl staining and the cellular layer in CA1, CA3, and DG region of the hippocampus of the WT and KO rats. **(I)** Neuron densities were calculated in the CA1, CA3, and DG region of hippocampus. All data are presented as mean ± standard error of the mean (SEM). (^∗∗∗^*p* < 0.001, ^∗∗^*p* < 0.01, ^∗^*p* < 0.05; ns., not significant, two-sided Student’s *t*-test was used).

In the present study, the CRISPR/Cas9 system was used to introduce deletions or mutations in exon 4 of the *Fmr1* gene in rats (**Figure 1A**). Sanger sequencing showed that one of the offspring lines carried a deletion of five amino acids and a G-A mutation in the *Fmr1* gene (**Figure 1B**). This genetic modification resulted in a frame-shift starting from the second Agenet-like 2 domain in FMRP (**Figure 1C**). RT-PCR analyses of the expression of the *Fmr1* transcript in the hippocampus and the cortex were conducted using three pairs of primers: one pair upstream of exon 4 of the *Fmr1* gene in rats and two pairs downstream of the gene. Similar to the expression of the *Fmr1* transcript in KO mice (Mientjes et al., 2006), expression of the *Fmr1* transcript in *Fmr1^exon4-KO^* rats was approximately 18.58–33.78% of that of the WT rats (**Figures 1D,E**). The primers targeting the sequence upstream of exon 4 yielded statistically significant increases in the transcript expression levels in the hippocampus that were slightly higher than those of the two primer pairs targeting regions downstream of exon 4 (**Figure 1E**). Western blotting, using specific anti-FMRP antibodies, confirmed the presence of FMRP in the cortex and hippocampus lysate of the WT [X*^Fmr1^*^(+)^Y] rats but not in that of the KO rats [X*^Fmr1^*^(-)^Y] (**Figure 1F** and **Supplementary Figure S1**, cortex: WT, 0.21 ± 0.02, KO, 0.06 ± 0.01, *n* = 3, *p* < 0.01; hippocampus: WT, 0.29 ± 0.03, KO, 0.03 ± 0.02, *n* = 3, *p* < 0.01). When X*^Fmr1^*^(+)^Y rats were crossed with X*^Fmr1^*^(+)^X*^Fmr1^*^(-)^ rats, 50.24% of the male offspring were KO rats (109 of 211 male animals from eight breeding pairs), which is consistent with the expected Mendelian ratio. The male KO rats showed normal development curves (**Figure 1G**). The brains of the KO rats exhibited normal histology of hippocampal and neuron densities (**Figures 1H,I**, CA1: WT, 2533 ± 163.3, KO, 2578 ± 164.8, *n* = 3, *p* > 0.05; CA3: WT, 1711 ± 145.7, KO, 1644 ± 104.2, *n* = 3, *p* > 0.05; DG: WT, 5289 ± 185.9, KO, 5467 ± 312.7, *n* = 3, *p* > 0.05), suggesting that FMRP deletion did not cause prenatal lethality or pervasive developmental deficits.

### Impaired Basal Synaptic Transmission and Synaptic Plasticity in *Fmr1* KO Rats

**FIGURE 2:**
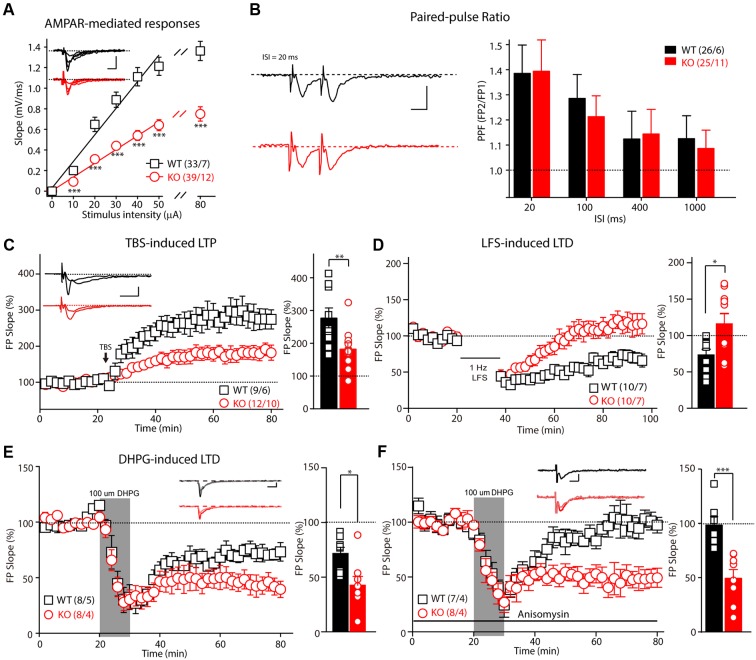
Impaired basic synaptic transmission and long-term plasticity in hippocampal CA3–CA1 synapses in *Fmr1* KO rats. **(A)** The α-amino-3-hydroxy-5-methyl-4-isoxazolepropionic acid receptor(AMPAR)–mediated field excitatory postsynaptic potential (fEPSP) and the input-output curve were reduced in 8-week-old *Fmr1* knockout (KO) rats. The initial slopes of the evoked fEPSP were plotted as a function of the stimulus intensity. **(B)** Left: Representative fEPSP traces from the control and *Fmr1* KO rats evoked by two consecutive stimuli with a 20-ms ISI. Right: Paired-pulse facilitation was normal in the 8-week-old KO rats. **(C)** The theta-burst stimulation (TBS)–induced long-term potentiation (LTP) was impaired in the 8-week-old KO rats. The inset (left panel) shows representative traces before and after LTP induction. The mean fEPSP slopes averaged 50–60 min after LTP induction in the wild-type (WT) and KO rats (Student’s *t-*test; right). **(D)** The low-frequency stimulus (LFS)–induced long-term depression (LTD) was impaired in the 8-week-old KO rats. **(E)** *Fmr1* KO rats showed an enhanced 3,5-dihydroxyphenylglycine (DHPG)–induced LTD (left: represented traces of WT and KO rats). **(F)** The protein synthesis inhibitor anisomycin blocks the DHPG-induced LTD of the WT rats but has no effect on the *Fmr1* KO rats. All data are presented as mean ± standard error of the mean (SEM). The scale bars represent 10 ms, 1 mV (^∗^*p* < 0.05, ^∗∗^*p* < 0.01, ^∗∗∗^*p* < 0.001; ns., not significant. The number of hippocampal slices (left) and rats (right) used in each experiment is indicated in parentheses).

Patients with FXS exhibit severe mental retardation that is caused by synaptic dysfunction. We first examined synaptic transmission and plasticity in the hippocampal Schaffer collateral pathway in acute slice preparation. Extracellular recordings were performed to monitor the fEPSP elicited by the stimulation of Schaffer collateral/CA1 glutamatergic fibers. The slope of the input-output curve of the α-amino-3-hydroxy-5-methyl-4-isoxazolepropionic acid receptor (AMPAR)–mediated fEPSP was statistically significantly decreased in the *Fmr1* KO rats compared with that of the WT littermate controls (**Figure 2A**, slope*_WT_* = 0.015 ± 0.001, slope*_KO_* = 0.009 ± 0.001, *p* < 0.001), demonstrating impairment of the basal synaptic transmission at the CA3–CA1 excitatory synapses. Next, we measured the synaptic facilitation induced by two identical stimuli separated by various intervals, which is an indicator of short-term plasticity. As shown in **Figure 2B**, paired-pulse facilitation was normal in the *Fmr1* KO rats when compared with that of the control rats. We next examined the effect of FMRP inactivation in CA3 neurons on TBS-induced LTP in the Schaffer collateral pathway. The amplitude of TBS-induced LTP (slope averaged 50–60 min post-TBS stimulation) was markedly impaired in the *Fmr1* KO rats (**Figure 2C**, WT: 277.8 ± 30.3%; KO: 183.4 ± 18.5%, *t* = 2.770, df = 18, *p* < 0.01), demonstrating a critical role of FMRP in regulating LTP at CA3–CA1 synapses. Therefore, we next asked whether the loss of FMRP in rats alters the maintenance of long-term depression. The LFS-induced LTD was statistically significantly reduced in *Fmr1^exon4-KO^* rats (**Figure 2D**, WT: 73.5 ± 7.2%; KO: 116.2 ± 13.7%, *t* = 2.752, df = 18, *p* < 0.05). The magnitude of the mGluRs-dependent LTD elicited by directly activating group I mGluRs with the agonist DHPG was statistically significantly greater in slices from the *Fmr1* KO rats compared with their control littermates (**Figure 2E**, WT: 71.7 ± 5.7%; KO: 42.7 ± 8.1%, *t* = 2.948, df = 14, *p* < 0.05). Moreover, treating the slice with anisomycin (20 μM), a protein synthesis inhibitor, prevented the maintenance of the mGluR-dependent LTD of the WT rats (98.38 ± 7.602%) but not that of the KO rats (49.49 ± 7.954%, **Figure 2F**). This result suggested that a DHPG-induced LTD does not require protein synthesis in *Fmr1^exon4-KO^* rats. Thus, FMRP differentially regulates the LTP and LTD in the hippocampal CA3-CA1 synapses in an induction-specific manner.

### *Fmr1* KO Rats Exhibit Altered Learning in the Morris Water Maze Test

**FIGURE 3:**
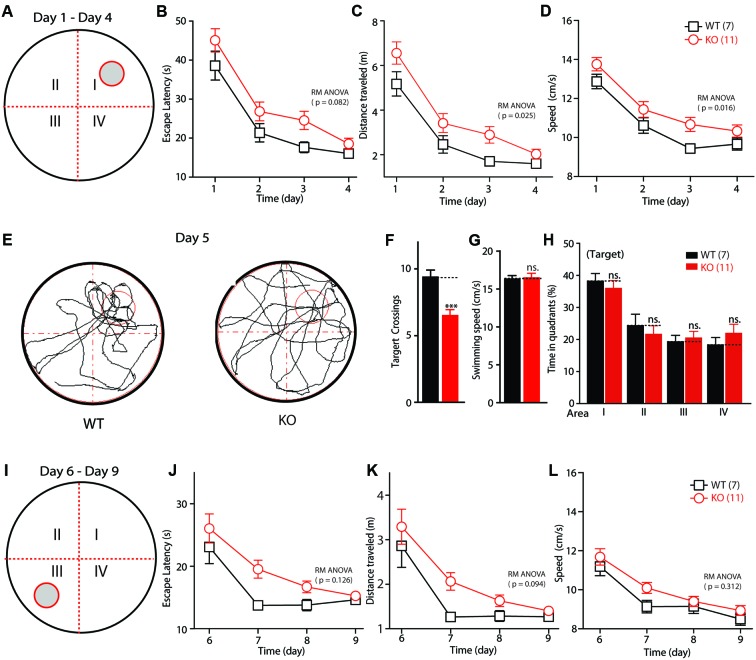
Impaired spatial learning and memory in *Fmr1* KO rats in the Morris water maze. **(A)** Schematic diagram of the apparatus. **(B)** Plot of escape latency. **(C)** Plot of travel distance. **(D)** Plot of swimming speed required for the rats to find the visible platform during the learning phase. **(E)** Representative movement traces. The empty red circles (left) indicate the location of the former platform, which was removed in the probe test. **(F)** Summary of the number of crosses of the former platform area. **(G)** Swimming speed in the probe test. **(H)** Time in each quadrant during the probe test (*p* < 0.001 target quadrant vs. Q2, Q3, or Q4). **(I)** Schematic diagram of the apparatus. The visible platform was placed in the third quadrant, which was opposite to that used during the learning phase (the first quadrant). **(J)** Plot of escape latency. **(K)** Plot of travel distance. **(L)** Plot of swimming speed required for the rats to find the visible platform during the reverse learning phase. All data are presented as mean ± SEM. Repeated-measures analysis of variance (RM ANOVA) was used to evaluate the difference between the two curves in **(A–D)** and **(J–L)**. Student’s *t*-test was used to compare the two groups **(F,G)**. *Post hoc* analysis was used to analyze the statistically significant ANOVA results **(H)** (^∗∗∗^*p* < 0.001; ns., not significant).

Altered *Fmr1* gene function is the major cause of mental retardation in patients with FXS. To test whether deletion of the *Fmr1* gene affects the spatial learning ability of KO rats, their performance was evaluated in a Morris water maze. The training time comprised 1-min periods over 4 days (**Figure 3A**). The escape latency and the travel distance of the WT and KO rats were statistically significantly reduced on days 1–4 (the training period) compared with these measurements on the test days [**Figures 3B–D**; escape latency: *F*_(3,48)_ = 64.655, *p* < 0.001; distance traveled: *F*_(3,48)_ = 64.941, *p* < 0.001; swimming speed: *F*_(3,48)_ = 45.650, *p* < 0.001]. Furthermore, in the KO rats, the escape latency was lengthened in comparison with that of the WT animals [**Figure 3B**, genotype *F*_(1,16)_ = 3.435, *p* = 0.082], the distance traveled before finding the hidden platform was longer [**Figure 3C**, genotype *F*_(1,16)_ = 6.083, *p* = 0.025], and swimming speed was statistically significantly increased [**Figure 3D**, genotype *F*_(1,16)_ = 7.186, *p* = 0.016]. After 4 days of training, the rats were tested in the same maze but without the platform (the probe test phase of memory; **Figure 3E**). As shown in **Figure 3F**, the KO rats crossed the target area statistically significantly less often than did the WT controls (WT: 9.43 ± 0.48, *n* = 7; KO: 6.55 ± 0.39, *n* = 11, *p* < 0.001); this difference was not due to impaired motor function, as the swimming speeds of the rats of each genotype did not differ statistically significantly (**Figure 3G**, WT: 16.38 ± 0.39, *n* = 7; KO: 16.55 ± 0.52, *n* = 11, *p* > 0.05). The time in each quadrant of the KO and WT littermate controls was not statistically significantly different (**Figures 3H,I**: WT: 38.17 ± 2.42, *n* = 7, KO: 35.99 ± 2.40, *n* = 11, *p* > 0.05; II: WT: 24.31 ± 3.60, *n* = 7, KO: 21.63 ± 2.64, *n* = 11, *p* > 0.05; III: WT: 19.25 ± 2.07, *n* = 7, KO: 20.46 ± 2.12, *n* = 11, *p* > 0.05; IV: WT: 18.26 ± 2.38, *n* = 7; KO: 21.92 ± 2.83, *n* = 11, *p* > 0.05). The WT and KO rats spent more time in the target quadrant. After the probe test phase, the rats were subjected to reverse learning, and the hidden platform was switched to the opposite quadrant to test behavioral flexibility (**Figure 3I**). The WT rats consistently found the new hidden platform more quickly than did the KO rats, but there was no statistically significant difference between the two groups [**Figures 3J–L**; escape latency: reversal training day, *F*_(3,48)_ = 24.566, *p* < 0.001; genotype, *F*_(1,16)_ = 2.605, *p* = 0.126; distance traveled: reversal training day, *F*_(3,48)_ = 27.113, *p* < 0.001; genotype, *F*_(1,16)_ = 3.160, *p* = 0.094; swimming speed: reversal training day, *F*_(3,48)_ = 60.115, *p* < 0.001; genotype, *F*_(1,16)_ = 1.089, *p* = 0.312]. These results show that deletion of FMRP in rats impaired their ability to obtain spatial learning and memory and to maintain a normal ability to retain memories.

### *Fmr1* KO Rats Display Impaired Social Interaction

**FIGURE 4:**
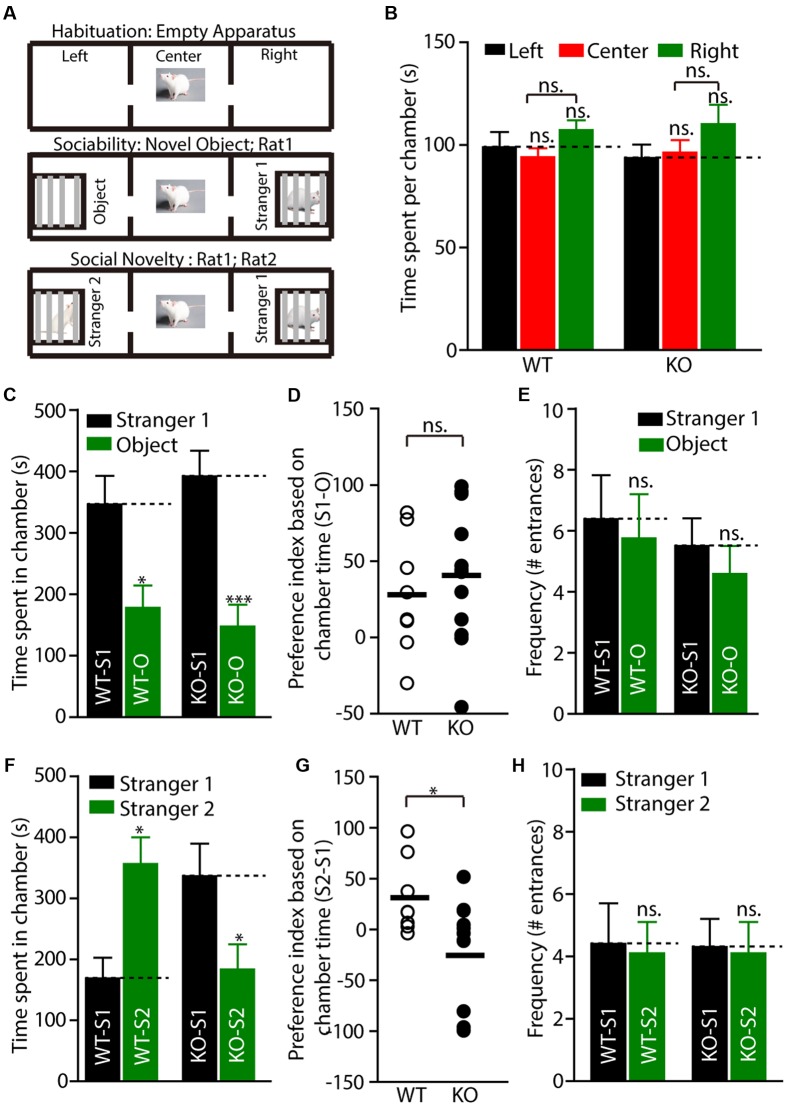
*Fmr1* KO rats display impaired social interaction in the three-chamber test. **(A)** Diagram of the testing apparatus with two outer side chambers, each housing a novel object or stranger rat behind perforated Plexiglas, and the center chamber where the subject was started. Two doorways allowed the subject to move freely between all chambers. **(B)** A WT or KO subject was allowed to explore the apparatus. The mean total duration each subject spent per chamber (including time at partitions within side chambers) is shown, WT: *n* = 8; KO: *n* = 12. **(C–E)** Quantification of the results in **(A)** (middle), as shown by the amount of time spent in chamber **(C)** with a novel rat (stranger1, S1) vs. an inanimate object (O), or the preference index derived from the numerical difference between the time spent in chamber **(D)** with S1 and O divided by total time spent × 100. Frequency of subject entry into each side chamber from the center chamber is shown **(E)**. **(F–H)** Quantification of the results in **(A)** (bottom). S2 (stranger2) and S1 (stranger1). All data are presented mean ± SEM. [^∗^*p* < 0.05, ^∗∗∗^*p* < 0.001; ns., not significant; one-way analysis of variance (ANOVA) and two-sided Student’s *t*-test were used].

Social dysfunction has been observed in patients with FXS and the animal models of FXS. To determine whether *Fmr1* KO rats display social deficits, we monitored the behavior of the rats in the three-chamber apparatus (Nadler et al., 2004), in which the social approach of a rat toward a stranger rat trapped in a wire cage can be measured. We first tested the WT and KO rats in three empty chambers; the two genotypes showed no difference (**Figures 4A,B**, WT: left: 98.86 ± 7.4, center: 93.9 ± 4.5, right: 107.2 ± 4.8, *p* > 0.05; KO: left: 93.7 ± 6.5, center: 96.2 ± 6.1, right: 110.1 ± 9.5, *p* > 0.05). In the sociability test, a novel object was placed in one side chamber, and a novel, same-sex rat (stranger1) was placed in the other side of the chamber. The WT and KO rats showed normal performance as measured by the amount of time spent in each chamber (**Figure 4C**, WT: stranger1: 346.3 ± 46.5 s, object: 177.9 ± 36.2 s, *p* < 0.05; KO: stranger1: 391.8 ± 41.7 s, object: 147.4 ± 35.5 s, *p* < 0.001), the preference index derived from these parameters (**Figure 4D**, WT: 28.06 ± 13.7, KO: 40.72 ± 12.8, *p* > 0.05), and the frequency of subject entry into each side chamber from the center chamber (**Figure 4E**, WT: stranger1: 6.4 ± 1.5, object: 5.8 ± 1.5, *p* > 0.05; KO: stranger1: 5.5 ± 0.9, object: 4.6 ± 0.9 s, *p* > 0.05). However, in the social novelty test, when the inanimate object was replaced with another stranger rat (stranger2), stranger1 was observed to be a familiar stimulus. The *Fmr1* KO rats spent less time with stranger2 compared to the amount of time spent with stranger1 (**Figure 4F**, WT: stranger1: 168.7.3 ± 34.0 s, stranger2: 356.0 ± 44.0 s, *p* < 0.05; KO: stranger1: 336.3 ± 53.2 s, stranger2: 183.5 ± 41.1 s, *p* < 0.05). The preference index derived from these parameters was also different (**Figure 4G**, WT: 31.23 ± 12.9, KO: -25.46 ± 15.4, *p* < 0.05), but the frequency of subject entry into each side chamber from the center chamber of the KO rats was similar to that obtained from the WT rats (**Figure 4H**, WT: stranger1: 4.4 ± 1.3, stranger2: 4.1 ± 1.0, *p* > 0.05; KO: stranger1: 4.3 ± 0.9, stranger2: 4.1 ± 1.0, *p* > 0.05). These results suggest that *Fmr1* KO rats are impaired in terms of social novelty recognition but display normal sociability or social anxiety.

### *Fmr1* KO Rats Demonstrate Normal Locomotor Activity and Normal Anxiety Levels

**FIGURE 5:**
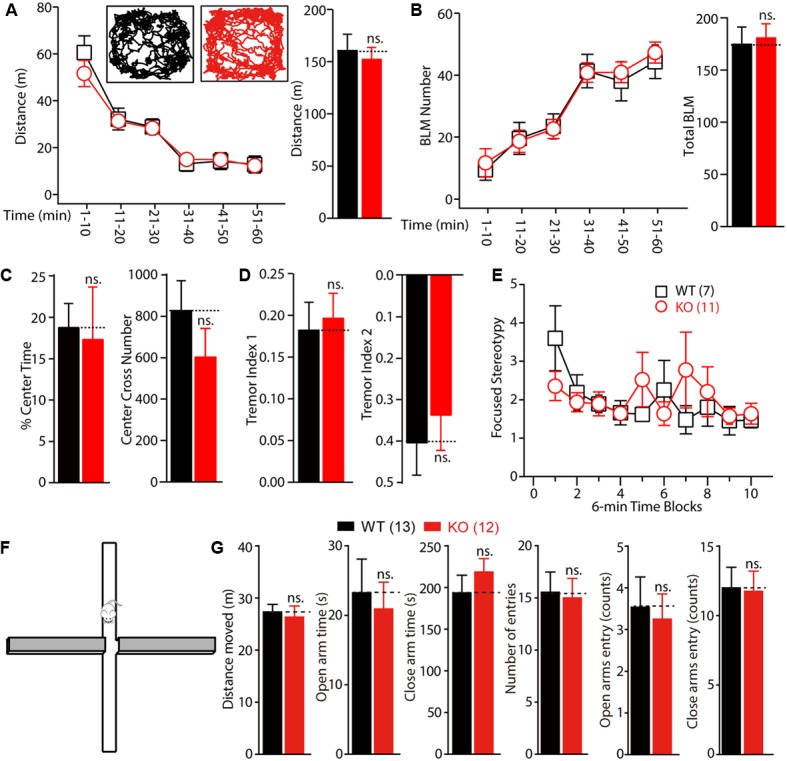
Normal locomotor activity and anxiety of *Fmr1* KO rats in force plate actometer and elevated plus maze test. **(A,B)** *Fmr1* KO rats showed normal exploratory behavior **(A)** and bouts of low mobility (BLM) **(B)** on the force plate. Rats were allowed to move freely on the force plate actometer for 60 min. Distance traveled and BLM were quantified every 10 min (left) and for the total 60 min period (right). The inset shows the representative movement trajectories of the WT and KO rats. **(C)** The percentage of time spent in the center field of the force plate and the number of crosses of the center field were measured. **(D)** KO rats showed normal whole-body tremor, which was quantified from the force variation data. **(E)** The focused stereotypy scores of the KO rats were similar to those of their WT littermate controls. The focused stereotypy scores were calculated from the intense rhythmic movement data of the rats on the force plate. **(F)** Diagram of the elevated plus maze test apparatus. **(G)** The distance the rats moved, in open and close arm time; there was no statistically significant difference in the open and close arm entries between the WT and KO rats. All data are presented as mean ± standard error of the mean (SEM). (ns., not significant, two-way ANOVA and two-sided Student’s *t*-test were used).

To test whether motor dysfunction might contribute to the learning deficits observed in the Morris water maze test, the locomotor activity of *Fmr1* KO rats was measured on a force-plate actometer (Fowler et al., 2001). The travel distances and the number of BLM of the KO and WT littermate controls were not statistically significantly different (**Figures 5A,B**, distance: WT: 133.25 ± 8.93 m, *n* = 7; KO: 136.64 ± 15.25 m, *n* = 11, *p* > 0.05; BLM: WT: 197.25 ± 20.45, *n* = 7; KO: 199.33 ± 19.9, *n* = 11, *p* > 0.05). Furthermore, the KO rats spent essentially the same percentage of time in the center area (23.2 cm × 23.2 cm; **Figure 5C** left, WT: 18.82 ± 2.83, *n* = 7; KO: 17.39 ± 6.26, *n* = 11, *p* > 0.05) and made a similar number of leaps over the center (**Figure 5C** right, WT: 830.43 ± 141.42, *n* = 7; KO: 601.64 ± 136.52, *n* = 11, *p* > 0.05). The tremor index of the KO and WT rats (calculated from the power spectra data using Fourier analysis) was also not statistically significantly different (**Figure 5D**, tremor index 1: WT: 0.18 ± 0.03, *n* = 7; KO: 0.17 ± 0.03, *n* = 11, *p* > 0.05; tremor index 2: WT: -0.41 ± 0.08, *n* = 7; KO: -0.30 ± 0.10, *n* = 11, *p* > 0.05). The frequency of stereotypical behavior declined over time after the WT and KO rats were placed on the force plate, but there were no genotypic differences [**Figure 5E**, genotype, *F*_(1,160)_ = 0.1110, *p* = 0.7395; time block, *F*_(9,160)_ = 1.310, *p* = 0.2354]. These results demonstrate that deletion of FMRP in rats had no detectable effects on motor function.

To explore whether the KO rats showed anxiety and hyperactivity, we performed the EPM test. The time spent in open arms and close arms were not different between genotypes (**Figures 5F,G**, open arms: WT vs. KO, 23.25 ± 4.85 s vs. 20.93 ± 3.83 s; close arms: WT vs. KO, 193.50 ± 21.09 s vs. 218.50 ± 16.09 s, WT: *n* = 13, KO: *n* = 12). These results indicated that the *Fmr1* KO rats showed normal anxiety level with the WT rats.

### *Fmr1* KO Rats Show Macroorchidism

**FIGURE 6:**
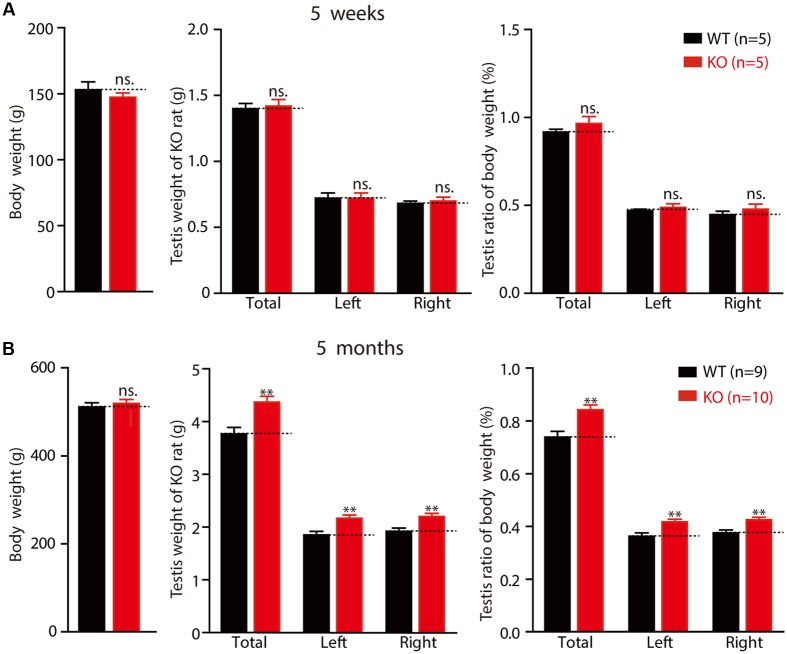
*Fmr1* KO rats exhibit macroorchidism. **(A)** *Fmr1* KO rats’ body weight, testis weight and testis ratio of body in 5-week-old *Fmr1* KO rats. **(B)** *Fmr1* KO rats’ body weight, testis weight and testis ratio of body in 5-month-old *Fmr1* KO rats. All data are presented as mean ± SEM (^∗∗^*p* < 0.01, two-sided Student’s *t*-test was used).

Macroorchidism, one of the hallmark symptoms experienced by patients with FXS, is also observed in several FXS animal models (Hamilton et al., 2014). Therefore, we examined the weight of the testes from 5-week-old to 5-month-old rats. As mentioned above, there were no differences in the average body weights of the WT and KO rats (**Figure 6A** left: WT 152.92 ± 6.10 g, *n* = 5; KO 147.26 ± 3.41 g, *n* = 5, *p* > 0.05; **Figure 6B** left: WT 511.26 ± 9.79 g, *n* = 9; KO 519.39 ± 8.89 g, *n* = 10, *p* > 0.05). No differences were observed in the testes of KO rats at the age of 5 weeks, in either net weight (**Figure 6A** middle: total: WT 1.4 ± 0.04 g, KO 1.4 ± 0.05 g, *p* > 0.05; left: WT 0.72 ± 0.04 g, KO 0.72 ± 0.04 g, *p* > 0.05; right: WT 0.68 ± 0.02 g, KO 0.70 ± 0.03 g, *p* > 0.05) or organ relative weight (**Figure 6A** right: total: WT 0.92 ± 0.02%, KO 0.97 ± 0.04%, *p* > 0.05; left: WT 0.47 ± 0.01%, KO 0.49 ± 0.02%, *p* > 0.05; right: WT 0.45 ± 0.02%, KO 0.48 ± 0.03%, *p* > 0.05). However, the net weight of the 5-month-old *Fmr1* KO rats’ testes was statistically significantly heavier than that of the WT rats (**Figure 6B** middle: total: WT 3.77 ± 0.12 g, KO 4.37 ± 0.11 g, *p* < 0.01; left: WT 1.85 ± 0.07 g, KO 2.17 ± 0.06 g, *p* < 0.01; right: WT 1.92 ± 0.06 g, KO 2.20 ± 0.06 g, *p* < 0.01), and the organ relative weight was also heavier than that of the WT rats (**Figure 6B** right: total: WT 0.74 ± 0.02%, KO 0.84 ± 0.02%, *p* < 0.01; left: WT 0.36 ± 0.01%, KO 0.42 ± 0.01%, *p* < 0.01; right: WT 0.38 ± 0.01%, KO 0.42 ± 0.01%, *p* < 0.01). These results demonstrate that *Fmr1* KO rats display macroorchidism at the age of 5 months.

## Discussion

In this study, we identified hippocampal physiology, hippocampal-dependent, and social behavior of *Fmr1* KO rats, which were generated by creating a five amino acid deletion in exon 4 of the *Fmr1* gene of a rat using the CRISPR/CAS9 method. FMRP consists of several protein domains: nuclear localization signal (NLS), two hnRNP-K-homology (KH) domains (KH1 and KH2), nuclear export signal (NES), and arginine-glycine-glycine (RGG box). FMRP regulates protein translation by binding to approximately 4% of the mRNA in the mammalian brain through the two KH domains and the RGG box (Ashley et al., 1993; Siomi et al., 1993). In the present study, the genomic modification caused a frame-shift, but not an in-frame deletion in exon 4, ensuring that the remaining transcript, if any, would not generate a truncated FMRP with functional domains (**Figures 1A–C**). Western blotting analysis confirmed the absence of FMRP in the brains of KO rats (**Figure 1F**). The *Fmr1^exon4-KO^* rats exhibited grossly normal development (**Figure 1G**) and normal locomotor activity, as reflected by normal activity in the open field test and the force plate tests and a normal anxiety level in the EPM test (**Figure 5**). These observations demonstrate that inactivation of the *Fmr1* gene in rats does not cause global motor dysfunction.

The normal function of the hippocampus, including LTP at the hippocampal Schaffer collateral pathway, is essential for learning during the Morris water maze test (Nosten-Bertrand et al., 1996; Lu et al., 1997; Moser et al., 1998; Jia et al., 2001). In *Fmr1* KO mice, LTP induced by either TBS or high-frequency stimulation remained unaltered at the hippocampal Schaffer collateral pathway (Godfraind et al., 1996; Zhang J. et al., 2009; Yun and Trommer, 2011; Bostrom et al., 2015), while the LTD was consistently enhanced in the *Fmr1* KO mouse and rat models (Godfraind et al., 1996; Huber et al., 2002; Li et al., 2002; Till et al., 2015). The dependence of the hippocampal LTP on FMRP had not been investigated in rats before this study. In the present study, electrophysiological analysis revealed that the TBS-induced LTP was severely reduced in hippocampal CA3–CA1 synapses, providing a plausible explanation for the learning deficits observed in the *Fmr1* KO rats (**Figure 2D**). Furthermore, basal synaptic transmission, as measured by the slope of the input–output curve, was statistically significantly reduced in the *Fmr1* KO rats, whereas short-term plasticity, a presynaptic phenomenon, was unchanged. These data imply that postsynaptic plasticity might be affected more severely than presynaptic plasticity at *Fmr1* KO synapses. The *Fmr1^exon4-KO^* rats exhibited an enhanced DHPG-induced LTD (**Figure 2E**), and this enhancement is independent of protein synthesis (**Figure 2F**), similar to previous studies of KO mice (Nosyreva and Huber, 2006; Till et al., 2015). Consistent with previous studies with another line of *Fmr1* KO rats, which was generated by SAGE Lab using ZFN technology to target intron 7 and exon 8 of *Fmr1* (Hamilton et al., 2014), both lines exhibited an enhanced DHPG-induced LTD, and social dysfunction in the three-chamber test (Till et al., 2015). Interestingly, *Fmr1^exon4-KO^* rats exhibited some distinct disease-related symptoms, including a reduced TBS-induced LTP (**Figure 2C**) and an LFS-induced LTD (**Figure 2D**) and learning disability in Morris water maze test (**Figure 3**). FMRP regulates the translation of the protein that is necessary for the induction and expression of synaptic plasticity and can impact synaptic plasticity through FMRP’s control of protein translation (Sidorov et al., 2013). Loss of FMRP may to some extent impact the interaction of the protein with AMPAR trafficking and then result in a reduced LTP and LTD through a postsynaptic mechanism.

In the Morris water maze test, an apparatus used to measure hippocampus-dependent spatial learning and memory (Schenk and Morris, 1985; Terry, 2009), *Fmr1^exon4-KO^* rats demonstrated slower learning and statistically significantly poorer performance during the probe test phase and the reversal leaning phase. These results are consistent with the fact that the majority of patients with FXS are diagnosed with a learning disability (Skinner et al., 2005; Hall et al., 2008). The probe trial difference in the *Fmr1^exon4-KO^* rats is intriguing and has not been observed in most of the *Fmr1* KO mouse lines. Studies using *Fmr1* KO mice have consistently revealed normal trial performance, whereas mixed results have been reported with respect to the memory acquisition and reversal learning processes (The Dutch-Belgian Fragile X Consortium, 1994; Kooy et al., 1996; D’Hooge et al., 1997; Paradee et al., 1999; Baker et al., 2010; Uutela et al., 2012). Results obtained using *Fmr1* KO mice do not correspond well with clinical observations of the symptoms of patients with FXS. Another explanation for differences in mouse and rat models of FXS might be differences in behavior-training paradigms, which seem to contribute to the performance difference in the probe trial. The *Fmr1^exon8-KO^* rats showed normal performance in the Morris water maze test when trained with an enhanced training paradigm (Till et al., 2015), suggesting that *Fmr1* KO rats may maintain spatial learning ability to some extent but have difficulty with complex spatial learning tasks. In the present study, we increased the training difficulty by hiding the platform underneath the water throughout the experiments, instead of using a visible platform as in the previous report (Till et al., 2015). Moreover, two *FMR1* paralogs, *FXR1P* and *FXR2P*, share a high domain homology with FMRP in mammals (Kaufmann et al., 2002). Functional compensation by *Fxr1p* and *Fxr2p* in the KO rats may also allow them to perform relatively well in easy tasks. Cooperation of FMRP and FXR2P in regulating synaptic plasticity has been observed in comparisons of *Fmr1* knockout, *Fxr2p* knockout, and *Fmr1/Fxr2p* double-knockout mice (Zhang J. et al., 2009). Therefore, it might be worthwhile to examine the cognitive ability and synaptic plasticity of *Fmr1-* and *Fxrps-compound* mutant rats. Thus, based on behavior and electrophysiological phenotypes, *Fmr1^exon4-KO^* rats constitute an ideal model with which to further explore the mechanisms underlying cognitive impairment in patients with FXS, which are directly related to the pathogenesis of FXS.

Patients with FXS exhibit abnormalities in social, communication, and stereotypic behaviors. In this study, *Fmr1^exon4-KO^* rats displayed normal social recognition but abnormal social novelty behavior in the three-chamber test. In the sociability test, the wild-type and *Fmr1^exon4-KO^* rats preferred to explore the first novel rat (stranger1) over an object relatively, and there was a lack of genotype effect. However, the *Fmr1^exon4-KO^* rats spent less time with the novel rat (stranger 2) in the social novelty test compared to the control rats, which is consistent with previous reports (McNaughton et al., 2008; Liu and Smith, 2009; Mines et al., 2010; Heitzer et al., 2013). These results are analogous to the abnormalities in individuals with FXS who display social withdrawal and anxiety (Demark et al., 2003; Cohen et al., 2005; Hatton et al., 2006). These results indicate that basal synaptic transmission in the Schaffer collateral pathway of *Fmr1^exon4-KO^* rats is deficit. The loss of long-term plasticity may constitute an essential mechanism in the Morris water maze test.

## Author Contributions

YT, CY, SS, YC, and XD contributed equally to this work. YT, CY, SS, YC, and XD carried out the experiments. JZ, FS, DZ, YL, GC, JL, QS, ZQ, and CZ contributed to the planning of the work. ZQ and CZ wrote the paper.

## Conflict of Interest Statement

The authors declare that the research was conducted in the absence of any commercial or financial relationships that could be construed as a potential conflict of interest.

## Supplementary Material

FIGURE S1 Full image of western blot.
