# Supplementary figures and images for: Loss of FMRP Impaired Hippocampal Long-Term Plasticity and Spatial Learning in Rats
Source: Front Mol Neurosci. 2017 Aug 28;10:269. doi: 10.3389/fnmol.2017.00269 (PMC5581399; doi:10.3389/fnmol.2017.00269)

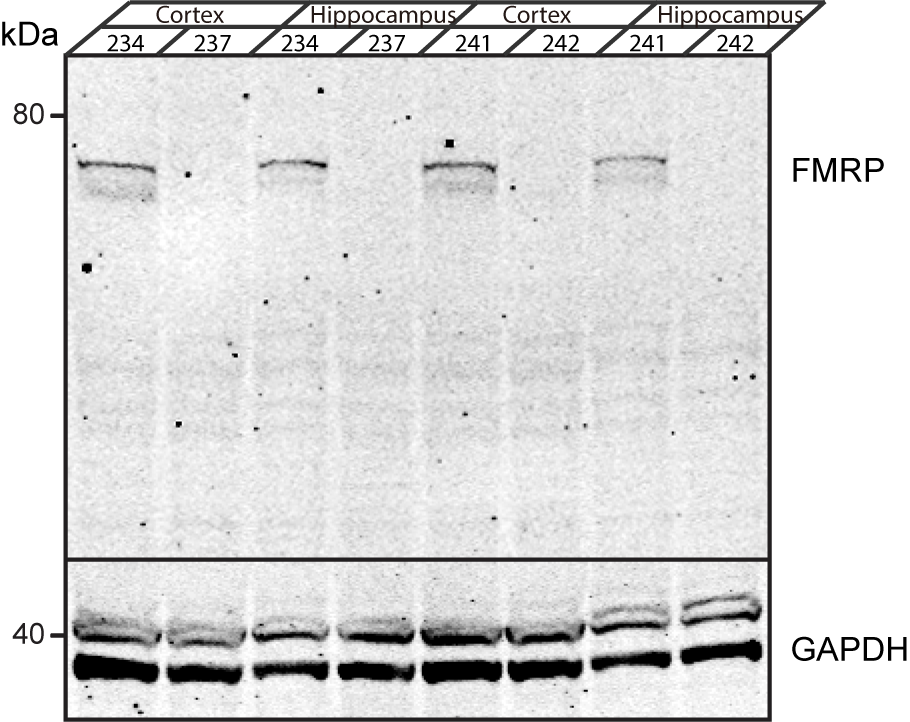

Supplement: FIGURE S1 — Full image of western blot. [file Image_1.TIF]
